# A Review of the Effects of Freshwater Habitat Quality on Aquatic & Semi‐Aquatic Turtles

**DOI:** 10.1002/ece3.74151

**Published:** 2026-08-11

**Authors:** K. Sullivan, Eric Nordberg, Deb Bower

**Affiliations:** ^1^ University of New England Armidale New South Wales Australia

**Keywords:** biomonitors, chelonians, environmental stressors, habitat loss, harmful algal blooms, pollution, salinity

## Abstract

Freshwater turtles and terrapins require an aquatic habitat as part of their natural ecology. The health of these habitats—the quality of the water, which can be affected by natural phenomena as well as pollution and human alterations of the watershed's natural flow—are inextricably intertwined with the persistence of the turtle species that rely on them. However, likely because of the vast number of species, habitats, and aquatic variables involved, a comprehensive review of the relationship between freshwater turtles and their aquatic environments has yet to be conducted. Here, we break down freshwater habitat quality into discreet natural and anthropogenic variables and summarize their impacts on the physical, behavioral, and population responses of freshwater turtles. We found that among natural variables, salinity and temperature were the most studied, while heavy metals received the most attention among the anthropogenic pollutants. Much of this research measured contaminant concentrations in turtle tissues, blood, or claws to assess their value as environmental biomonitors, rather than directly evaluating short‐ or long‐term health effects. Meanwhile, the impacts of many other natural water quality metrics such as pH, turbidity, and contaminants like coal and oil spills remain severely under‐studied. Taxonomic coverage was similarly biased towards a small number of common species, despite many threatened species being especially vulnerable to habitat degradation. By drawing attention to these gaps and biases, we hope to make the path clearer for future research to identify the impacts of habitat quality metrics on the health and distribution of freshwater turtles.

## Graphical abstract

This literature review aims to provide a comprehensive review of the relationship between freshwater turtles and their aquatic environments. We also highlight areas which have been over‐ or under‐studied and offer recommendations for future research.

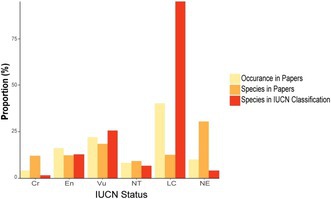

## 1 Introduction

Although freshwater systems only cover 2.3% of the Earth's surface, they are home to 9.5% of all recognized animal species, making them global hotspots for biodiversity (Balian et al. 2008). So long as these systems remain healthy, they can maintain their structure and function over long periods of time and through periods of extreme stress (Costanza and Mageau 1999). One of the ways freshwater ecosystem health is measured is through water quality; indices that quantify physical, chemical, and biological parameters, as well as anthropogenic additives (Baker 2005).

In the Anthropocene, industrial pollution, urban development, and agricultural runoff are leading to water quality degradation throughout the world's freshwater ecosystems (Akhtar et al. 2021). Common problems include build up of harmful chemicals, salinization, increasing temperatures, eutrophication, and harmful algal blooms (HABs) (Brooks et al. 2016; Amoatey and Baawain 2019; Suresh et al. 2023). Some of these variables are inextricably linked; for example, the increasing frequency and severity of HABs in freshwater and marine systems is tied to an exponential increase in nutrients from agricultural activities (Sellner et al. 2003).

These threats have long been associated with declines in the health of freshwater ecosystems. Poor water quality is linked to population declines in fish, freshwater invertebrates, amphibians, and birds (Cumberlidge et al. 2009; Chama and Siachoono 2015; Reid et al. 2019). This may be most obvious (and well‐studied) in fish populations, where oxygen depletion, eutrophication, and acidification have led to mass fish die‐offs (Brown et al. 1983; San Diego‐McGlone et al. 2008; Demeke and Tassew 2016; Baladia et al. 2025). Amphibians are also negatively impacted by pollution and declines in water quality, particularly through the accumulation of agricultural chemicals like pesticides, increased risk of disease, and declining species richness (Battaglin et al. 2016; Calderon et al. 2019). Likewise, aquatic reptiles are bound to be affected by reductions in the health of their environment; for example, Nile crocodile ( *Crocodylus niloticus* Laurenti, 1768) populations in the South African Olifants River catchment were attributed to years of industrial and agricultural pollution in the water system (Ashton 2010).

Among the many taxa affected by the Anthropocene are turtles; today 51.3% of the 357 recognized species are considered Threatened, Endangered, or Critically Endangered by the IUCN (Lovich et al. 2018; Rhodin et al. 2018; Stanford et al. 2020). Of these, 305 are non‐marine turtles, one of the most endangered groups in the world (Buhlmann et al. 2009). Common threats to turtles include invasive species, habitat loss, and over‐harvesting for food, medicine, or the pet trade (Stanford et al. 2020). In captivity there are clear links between water quality and turtle health (Wappel and Schulte 2004; Rangel‐Mendoza et al. 2014) so understanding how health of turtles relates to water quality in wild populations is paramount to predicting and avoiding future declines. Moreover, the life history attributes of many freshwater turtle species of delayed growth (Congdon et al. 2013) and long lives with low adult mortality rates (da Silva et al. 2022) suggest that as a taxa, the influence of the effects of reduced water quality on the demography of populations may be profound (Otten et al. 2023; Auge et al. 2024) and yet hidden by the long lives and cryptic nature of many species (Spencer 2018) or by nuanced physiological responses. For example, age‐specific population declines are probable with parameters where smaller animals (usually younger) are more sensitive such as the effect of increased salt concentration (Dunson and Heatwole 1986) and this may be difficult to detect through a study of demography alone (Ashley et al. 2021). A full synthesis of the realized and potential effects of water quality on freshwater turtles has not yet been compiled, but may be valuable to help detect trends among species, and to guide future research.

To that end, we synthesized existing research that evaluated freshwater turtle ecology within the context of their aquatic environment to determine (1) how turtles respond to changes in their aquatic environment, and (2) what research trends and gaps exist in the topic of freshwater turtle sensitivity to water quality. In particular, we highlighted areas that have been over‐ or under‐studied and offered directions for future research in this field.

## 2 Methods

### 2.1 Literature Search

We took a multi‐database approach to identify primary literature examining the impact of water quality on freshwater turtles. Papers were extracted from three databases: Web of Science, Scopus, and Google Scholar. Searches were conducted from 13 to 28 of April 2023, and again from 25 November to 2 December 2024, and included all existing papers written in English. The search terms used were chosen according to relevance. Cyanobacterial blooms are the most frequently occurring and severe type of algal blooms in freshwater systems, and microcystins are the most common cyanotoxin produced by these blooms (Landsberg 2002; Catherine et al. 2013; Gatz 2020). Therefore, for literature relating to algal blooms, we searched a combination of terms: “freshwater turtle*” AND “algal bloom*” OR “cyanobacteria” OR “cyanotoxin” OR “microcystin” “freshwater turtle*.” In addition, we selected six of the most common water quality parameters for further searches: “freshwater turtle*” AND “salinity” OR “temperature” OR “turbidity” OR “pH” OR “nitrogen” OR “dissolved oxygen,” as well as the general term “water quality” (Uddin et al. 2022). These terms were chosen to cover the broad variety of environmental variables that freshwater turtles encounter and adapt to in their natural habitats. Finally, to assess anthropogenic factors, we searched for the terms: “pollution” OR “pesticide” OR “herbicide” OR “industrial” OR “heavy metal*” OR “PCB” AND “freshwater turtle*.” These search terms were chosen as representative of some of the most common freshwater pollutants.

### 2.2 Selection Process

Each term was first searched in Web of Science, then Scopus, then Google Scholar. For the Web of Science and Scopus, all results were screened by reading article titles and abstracts. For Google Scholar, the first ten pages of results were screened by reading article titles and abstracts, with ten papers per page for a total of 100 papers per search term. This cut‐off was selected as very few relevant results were found after the first five pages; doubling that number minimized the exclusion of relevant results while providing a reasonable search parameter. All research articles that provided primary or secondary data, including laboratory or field‐based studies and computer models, were accepted. Studies that summarized or discussed compilations of pre‐existing data, such as literature reviews and opinions, as well as postgraduate theses and government reports, were excluded in this first round.

Once screened, all selected articles were read in full. To be included in our review, the articles must have examined at least one species of freshwater turtle in conjunction with at least one aquatic variable, the category of which included aquatic characteristics, quality, and natural and anthropogenic stressors. Results that met only one of the two search criteria (i.e., did not include at least one freshwater turtle species or which did not examine the effects of at least one specific aquatic characteristic on that species) were excluded from further analysis. Literature reviews were excluded on the basis that they did not present new information in this area of research. Papers in which the mode of exposure to variables was not aquatic (e.g., injections, topical applications to eggshells, incubation in substrate) were not included in further analysis. Papers studying over‐winter brumation in anoxia tolerant turtle species (e.g., Graham and Guimond 1995) were also excluded, on the basis that the lack of oxygen is a physiological response to a natural behavior rather than an environmental variable.

### 2.3 Data Extraction and Additional Information

A total of 1060 papers were initially returned using the specified search parameters (596 from Web of Science, 304 from Scopus, and 160 from Google Scholar). After filtering these for duplicates and papers that did not meet the inclusion criteria, we were left with 185 suitable papers (See Data S1). The country in which the study was conducted and the species studied were recorded for each paper. The IUCN conservation status of each species (Least Concern, Near Threatened, Vulnerable, Endangered, or Critically Endangered) was determined from the IUCN Redlist website and cross‐referenced with recent literature which included species assessed by the Tortoise and Freshwater Turtle Specialist Group (Rhodin et al. 2018, https://www.iucnredlist.org/). Then, each paper was categorized by one of the 13 environmental variables: six natural aquatic characteristics (salinity, temperature, turbidity, pH, dissolved oxygen, nitrogen), six anthropogenic pollutants (agricultural, industrial, polychlorinated biphenyls (PCBs), heavy metals, sewage wastewater, and oil or coal spills), and HABs. Agricultural pollutants included herbicides, pesticides, and fertilizers, and industrial pollutants included medical waste, per‐ and polyfluorinated substances (PFAS), and wastewater from manufacturing plants.

## 3 Results and Discussion

### 3.1 Study Species and Locations

**FIGURE 1:**
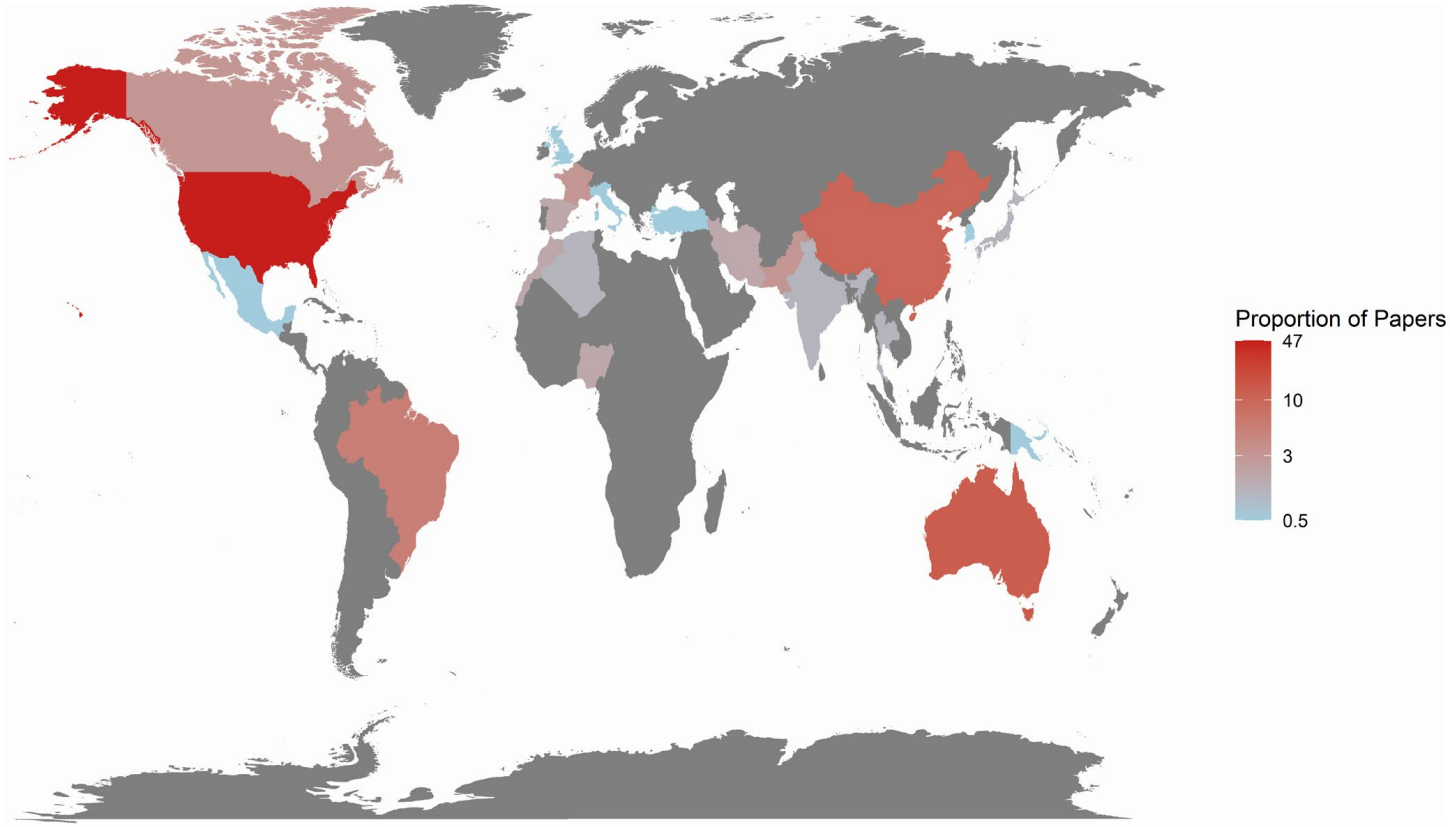
A gradient map demonstrating the proportion of the locations studied in the collected literature. Countries where no research was conducted are colored in gray.

The selected papers covered 58 freshwater turtle species in 23 countries (Figure 1). The greatest percentage of studies occurred in the United States of America (USA), accounting for 46.3% of all results; the next closest, comparatively, was Australia (13.6%). The USA is home to about 18%–20% of all freshwater turtle species, and the south‐eastern United States is considered a hotspot for turtle species richness and diversity, with 21 endemic species found in the Mississippi Valley alone (Ernst 2023). However, other “turtle hotspots” were severely underrepresented in the selected literature. For example, only 5.3% of studies were conducted in Brazil, despite the Rio Negro drainage containing equal richness of endemic species as the south‐eastern United States (14–16 species) (Buhlmann et al. 2009). India accounted for only 1.1% of locations studied in our review, while the Ganges–Brahmaputra River basin drainage of India and Bangladesh is considered the greatest turtle richness area in the world with 19 recognized endemic species (Buhlmann et al. 2009; Mittermeier et al. 2015). These hotspots are vulnerable to degradation via pollution and habitat destruction (Castello et al. 2013; Sinha and Loganathan 2015; Camara et al. 2019) and yet they are underrepresented in the literature compared to places like the United States. Even within the US, despite the high biodiversity of turtle species found in the Mississippi River Basin, the majority of papers focused on the common snapping turtle (*Chelydra serpentina*, Linnaeus 1958) and the painted turtle (*Chrysemys picta*, Schneider, 1783), two of the most common species in the United States and a poor representation of the region's phylogenetic diversity (Mittermeier et al. 2015; Thomson et al. 2021; Rhodin et al. 2025). Moreover, while some studies may be applicable across closely‐related taxa, it would be unreasonable to expect studies conducted on one group to be broadly applicable to all turtles. Therefore, there are obvious geographic regions of priority for understanding the effects of water quality on freshwater turtles.

The most studied species was the red‐eared pond slider *Trachemys scripta* (Thunberg and Schoepff, 1792), of which two of the three subspecies, *T. scripta scripta* (Schoepff, 1792) and *T. scripta elegans* (Wied, 1839), were specifically identified in the literature. *T. scripta* is a common species in the central and southern United States of America and has been introduced on every inhabited continent through the pet trade and as ornamental additions to ponds and fountains (Ficetola et al. 2012). Because of the species' wide distribution both inside and outside its native range, it is one of the most extensively studied species of any freshwater turtle (Seidel and Ernst 2006). This holds true for literature concerning water quality, where *T. scripta* was studied in 18.1% of publications. Interestingly, phylogenetic differences between turtles in different continents is likely to lead to differing tolerances and effects on species but the spread of *T. scripta* may provide a unique opportunity for a standardized model to compare the effects of water quality in different geographic regions, as invasive and exotic species are generally subject to less protection than native species in areas where it is invasive (Semenov 2010).

**FIGURE 2:**
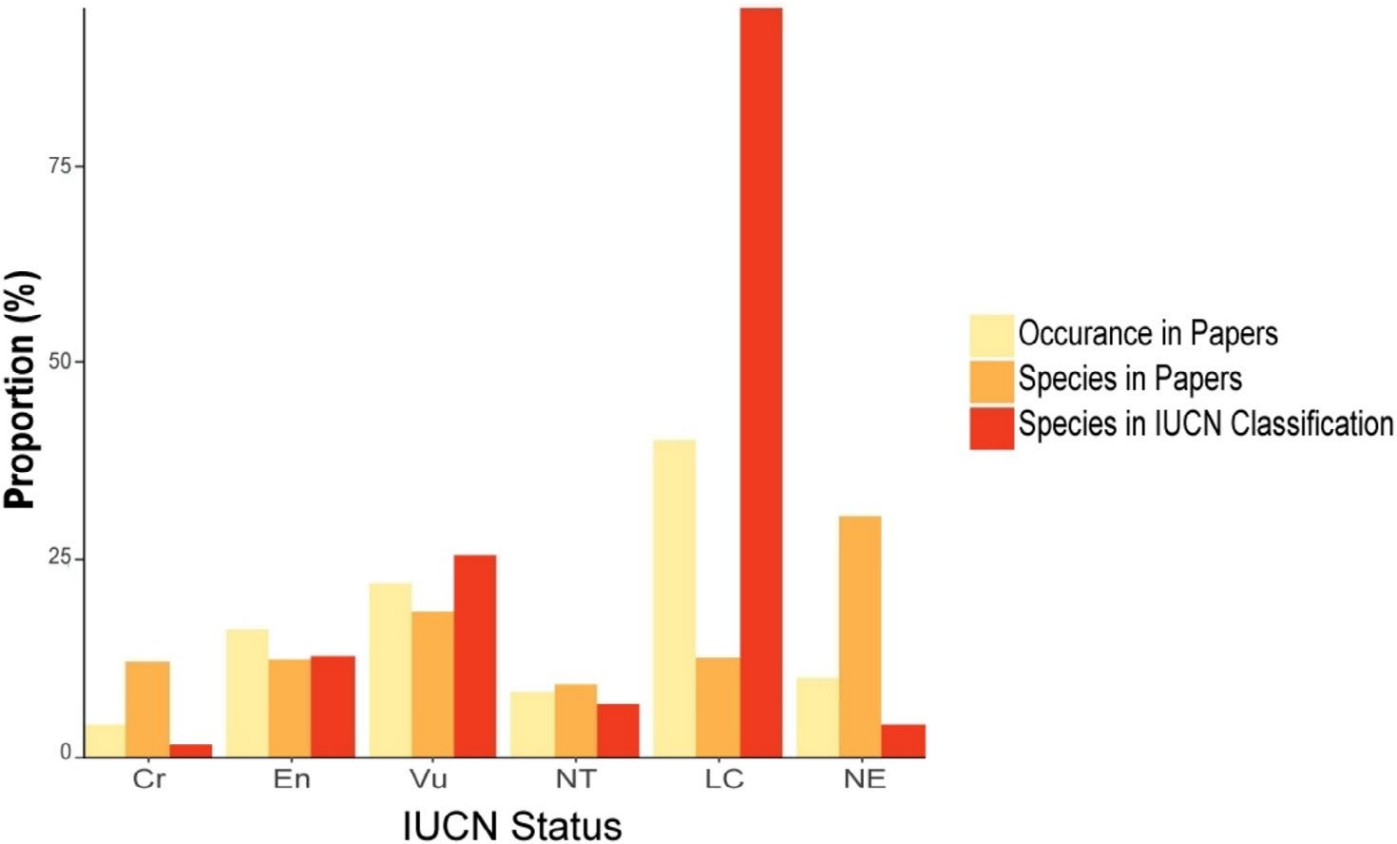
The proportion of turtle species in each classification as distributed by the designation by the IUCN as of 2018, the presence in selected papers and the distribution of designation among the 58 studied species. CR, critically endangered; EN, endangered; LC, least concern; NE, not evaluated; NT, near threatened; VU, vulnerable.

In addition to its dominance in the literature, *T. scripta* as a species of Least Concern is a representative of a much larger issue; chelonian species classified as Least Concern and Near Threatened were overrepresented compared to more endangered species. Excluding species that were not evaluated for conservation status, the percentage of papers that had species classified as Least Concern (87%) dwarfed the percentage of all those classified as Vulnerable, Endangered, and Critically Endangered combined (32.3%). Perhaps unsurprisingly, Critically Endangered species were the least studied, representing only 3.9% of species and 1.4% of papers, despite making up 11.9% of all species currently classified by the IUCN (Figure 2). This is in sharp contrast to the reality of the conservation status of turtles as a group, of which 20% are Critically Endangered and 15.6% are Endangered, and 16.1% are vulnerable (Rhodin et al. 2018). While studying threatened species may be more challenging than for common species, it is the threatened species that are at much greater risk of disappearing as a result of changing habitats, declining water quality, and pollution, and so should be a much higher research priority. To address the lack of research in the most threatened species, hatcheries, farms and head‐starting programs that produce an excess of juvenile animals, and zoos and aquariums that support conservation measures, may provide an opportunity to combine critical scientific questions necessary to identify threats, with conservation programs that are managing species. The increased collaboration and capacity of conservation programs to experimentally test attributes of water quality could vastly benefit our knowledge of freshwater turtles in relation to threats to wetland and river health.

A total of nine publications specifically examined the effect of harmful algal blooms or toxins produced by blooms on freshwater turtles. These papers were published between 2008 and 2021, and all but two studies were conducted in the United States of America; the other two were in Algeria and China, respectively. All studies focused on the physiological effects of algal bloom exposure, including toxicity symptoms and immune response.

**FIGURE 3:**
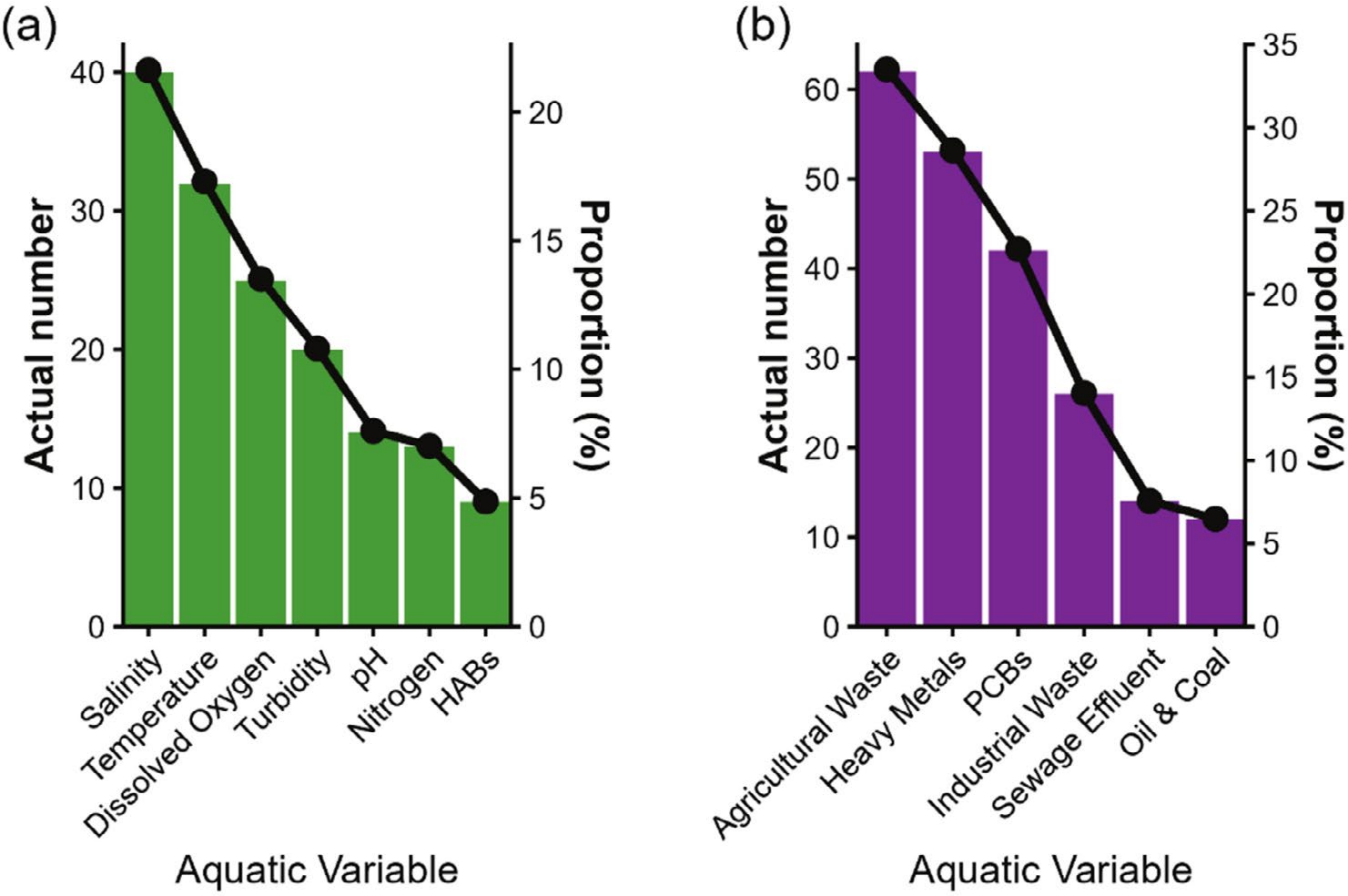
(a) The number of papers and the proportion of water quality parameters present in the collected literature. (b) The number of papers and the proportion of anthropogenic aquatic variables present in the collected literature. HABs stands for harmful algal blooms, and PCBs stands for polychlorinated biphenyls, an industrial chemical compound. On both graphs, the colored bars indicate the number of papers while the black line indicates the proportion present from the total collected literature.

Of the six natural water quality variables identified, salinity and temperature were the most studied (19.5% and 15.3% of studies, respectively) (Figure 3a). Publications measuring nitrogen yielded the fewest results, considered in only 13 of 185 (7%) papers. A majority of the studies on anthropogenic factors examined the concentrations of various pollutants in freshwater turtle tissues, claws, and blood, primarily to determine their viability as living environmental monitors as opposed to monitoring potential short‐ or long‐term impacts on health, behavior, or population trends. Heavy metals were the most studied of the six anthropogenic factors, accounting for 20% of papers retrieved, and followed closely by agricultural chemicals such as pesticides and herbicides (19%) and PCBs (15.3%) (Figure 3b). Other pollutants such as industrial waste, sewage, and oil and coal spills were under‐studied, despite the widespread damage they can cause to freshwater ecosystems (Schoonbee et al. 1995; Walsh et al. 2005; Wright et al. 2017). See Table 1 for a summary of the major findings, gaps, and suggested future research directions for the effects of natural and anthropogenic water quality variables on freshwater turtles. Overall, existing research on the effects of natural aquatic characteristics and human pollutants is remarkably uneven, leaning heavily towards utilizing freshwater turtles as indicators for the health of an environment without considering the impacts to the turtles themselves.

**TABLE 1:** A summary of the major findings, knowledge gaps, and recommended future research directions for natural and anthropogenic water quality parameters.

Water quality variable	Current state of knowledge	Major knowledge gaps	Recommended future research
HABs	Evidence that exposure to certain HAB toxins, including brevetoxin, leads to neurological symptoms & depressed immune response; mass mortality events have been correlated with HABs, but direct causation has not been confirmed	Very few papers examining the health effects of cyanobacterial HABs; there is no current evidence showing that turtles naturally exposed to HABs experience any serious health effects	Controlled exposure to cyanotoxins to document short‐ and long‐term health effects, and recovery/mortality rates; continued monitoring of populations repeatedly exposed to HABs for health & demographic effects
Natural Water Quality	Studies show mixed ability to adapt to increases in salinity, depending on species; increased temperatures may cause increases in basking time & decreased diving time; hypoxia may also lead to decreased diving time in bimodally respiring species	Very few studies on the effects of pH, turbidity, or nitrogen on health, behavior, or population demographics/range; there were also very few studies on the effects of cold‐water exposure	Inclusion of pH, turbidity, and nitrogen measurements when studying freshwater turtle habitat characteristics; controlled experiments examining the effects of temperature on juvenile growth rates; expansion of salinity studies to species outside the US which may be vulnerable to salinization of their habitats
Anthropogenic Pollutants	Most studies utilized turtles as biomonitors, taking claw & soft‐tissue samples to determine environmental contamination; also evidence showing heavy metal and PCB contamination can be transferred from mother to egg; some studies showed chemical‐specific effects on health (e.g., pesticide exposure led to depressed immune responses); certain species may also be adapting to live in sewage wastewater, as some populations were documented with much higher body size & condition than average	Very few studies on the effects of specific chemical contaminants (e.g., PCBs, pesticides, herbicides) on turtle health or population demographics; it is also unclear why certain species were more adapted to live in sewage effluent, or whether they experienced any long‐term health or demographic effects	Continued monitoring of populations living in sewage effluent for health & demographic changes; potentially controlled experiments exposing turtles to various chemical pollutants to determine immediate health effects & recovery rates
Oil & Coal	Some mass mortality events due to oil spills, but long‐term monitoring of populations have shown almost full recovery; no current evidence to suggest health impacts from coal ash spills	Limited studies on the immediate health impacts of exposure to oil, or long‐term health effects from coal ash spills which may also contain heavy metals	Due to the sporadic nature of coal and oil spills, in situ events are difficult to study; populations known to be exposed should continue to be monitored for health and demographic effects

Abbreviations: HABs, harmful algal blooms; PCBs, polychlorinated biphenyls.

### 3.2 Effects of Algal Blooms

Although harmful algal blooms are commonly recognized as threats to freshwater ecosystems (Reid et al. 2019), there is very little research on how they impact freshwater turtle species. A number of toxins have been associated with algal blooms; in freshwater blooms, 11 were identified as causing illnesses or even death in macroinvertebrates, fish, birds, domestic animals, and humans (Paerl et al. 2001; Landsberg 2002; Hallegraeff 2004). However, of the nine papers identified as researching the impacts of HABs on turtles, only four actually examined the physiological effects of HAB toxins. Another two merely recorded the concentrations of toxins in already deceased turtles, and the last two recorded algal blooms as potential factors affecting the distribution and demography of turtle populations.

Brevetoxins are neurotoxins caused primarily by the dinoflagellate *Karenia brevis* (Landsberg 2002). Because *K. brevis* is a marine species, freshwater species have little opportunity for exposure. However, brevetoxins can be transported inland via aerosolization from wave activity, and mortality in freshwater amphibians, as well as terrestrial mammals, has been attributed to brevetoxicity via this route of exposure (Kirkpatrick et al. 2010; Buttke et al. 2018). It is therefore plausible to deduce that freshwater turtles could be at risk of brevetoxin exposure, despite the exclusivity of brevetoxin‐producing blooms to marine ecosystems.

To study the effects of brevetoxins on turtles, *T. scripta* and *Malaclemys terrapin* were used as model species. Oral and intratracheal exposure caused neurological symptoms including head bobbing, ataxia, paralysis, and coma (Cocilova et al. 2017). In both modes of administration, brevetoxins were distributed through all systems and occurred in highest concentrations in the kidney and liver. However, all toxins were completely cleared from the turtles' bodies 24 h post treatment. These findings indicate some tolerance of freshwater turtles to brevetoxicity and an ability to recover from exposures.

In addition to neurological symptoms, brevetoxins may impact the immune health of turtles. Brevetoxin exposure, both oral and intratracheal, led to a depressed immune system and inflammation in *T. scripta* and *M. terrapin* , as well as markers of oxidative stress (Walsh et al. 2019). These results were consistent with those noted in marine turtles (Walsh et al. 2010; Perrault et al. 2017). In addition, brevetoxins and cyanotoxins are linked to tumor growth in marine turtles (Landsberg et al. 1999; Arthur et al. 2006; Perrault et al. 2017). Currently there is no evidence to support a correlation in tumor growth in freshwater turtles and exposure to *K. brevis* or cyanobacterial blooms. However, in both experiments examining the effects of brevetoxins, turtles were only exposed for the duration of one bloom (Cocilova et al. 2017; Walsh et al. 2019). In their natural occurrence, HABs can recur on an annual basis, leading to repeated exposure (Isles et al. 2015; Mishra et al. 2019; Hou et al. 2022). As of yet, we have no information on what the cumulative effects of HAB toxins may be.

While freshwater turtles are likely to be limited in their exposure to brevetoxins, the harmful algal blooms produced by cyanobacteria pose a very real potential threat. Under eutrophic conditions, many cyanobacteria form thick, brightly colored mats on the water's surface, known as harmful cyanobacterial blooms (Catherine et al. 2013). These mats can cause deoxygenation of water bodies and a significant increase in phytoplankton and algae biomass which often outcompete other primary producers and limit the growth of zooplankton, drastically reducing the overall productivity of the ecosystem (Robarts et al. 2005; Havens 2008). Many cyanobacteria also produce toxins that are associated with large‐scale illnesses or mortalities in fish, birds, and mammals (Carmichael 2001; Huisman et al. 2018; Falfushynska et al. 2023). In addition, it is well documented that marine turtles exposed to cyanobacteria are at increased risk of developing tumors (Landsberg et al. 1999; Arthur et al. 2006, 2008).

Harmful algal blooms may also cause diseases in freshwater turtles; ingestion of vegetation colonized by an unknown cyanobacteria caused neurologic dysfunction, diffuse vacuolation, and inflammatory lesions throughout the brain in *C. picta* (Mercurio et al. 2014). These symptoms are consistent with those observed in birds afflicted with vacuolar myelinopathy caused by cyanotoxicity (Wilde et al. 2005). Previously, vacuolar myelinopathy associated with HABs has only been described in birds and was even referred to as avian vacuolar myelinopathy (Wilde et al. 2005; Breinlinger et al. 2021); this study on *C. picta* represented the first evidence of the disease in response to cyanobacteria in another taxa.

There are a number of toxins associated with cyanobacteria blooms; the most prevalent are microcystins (Bláha et al. 2009; Ferrão‐Filho and Kozlowsky‐Suzuki 2011). This group is highly toxic, attacking the liver and causing cell death and internal bleeding in domestic and wild mammals, as well as humans (Landsberg 2002; Malbrouck and Kestemont 2006; Bláha et al. 2009). However, only one freshwater turtle mass mortality event, in Algeria, has been attributed to microcystins (Nasri et al. 2008). In the carcasses of the two species tested, *Emys orbicularis* (Linnaeus, 1758) and *Mauremys leprosa* , concentrations of microcystins were highest in the liver and vascular tissue. However, because only four of the 12 turtle carcasses were tested for toxins, and the study design was correlative in nature, it is difficult to conclusively attribute their deaths to the presence of microcystins. Similarly, Lake Taihu in China has a history of regular microcystis blooms, and toxins in *Pelodiscus sinensis* caught from there were most highly concentrated in the liver (Chen et al. 2009). In both studies, authors theorized that exposure occurred through the diet as opposed to inhalation or direct ingestion of algae. *Emys orbicularis* and *P. sinensis* are carnivorous, feeding primarily on invertebrates, tadpoles, and small amphibians (Kong et al. 2022). Because they were exposed through trophic accumulation, the concentration of microcystins in their tissues was lower than other examined species like *M. leprosa* , which is omnivorous and likely accumulated microcystins via direct consumption of filamentous algae (Bertolero and Busack 2017). However, it is unclear whether microcystin build up is fatal to freshwater turtles, or whether exposure leads to comorbidities. Notwithstanding this, several experiments on fish exposed to long periods or high concentrations of microcystins indicate that buildup can be extremely detrimental or even fatal so better understanding the effects on turtles is an important research direction (Tencalla et al. 1994; Jos et al. 2005; Acuña et al. 2012).

Exposure to microcystis blooms can impair the production and function of immune cells in humans, mice, birds, and fish (Diez‐Quijada et al. 2021), but only one study has been conducted on freshwater turtles. *C. picta* living in microcystin contaminated areas had a higher bactericidal capacity compared to those in an area with no detectable levels of the toxin, indicating that innate immune activity was elevated (Refsnider et al. 2021). However, tests of adaptive immune functioning in the same animals found no difference between exposed turtles and controls. This discrepancy may be due to seasonality and innate physiology; both hibernation and reproductive cycles influences immune activity in turtles (Schwanz et al. 2011). These factors should therefore be taken into careful account in future studies examining the effects of microcystins on freshwater turtles.

Though there is a dearth of data on the effect of algal blooms on freshwater turtles, there exists a wealth of information for other organisms. Mass deaths or long‐term health effects in marine turtles, dolphins, and manatees have been attributed to blue‐green algae (Foote et al. 1997; Fire et al. 2015; Foley et al. 2019). In particular, marine turtles exposed to cyanotoxins exhibited an increase in tumorous growths—a phenomenon also reported in several mammal species, including humans (Landsberg et al. 1999; Zanchett and Oliveira‐Filho 2013; Perrault et al. 2017).

More broadly, algal blooms are also associated with declines in community species richness and abundance across many aquatic plant and animal taxa (Kvitek and Bretz 2005; Gannon et al. 2009; Bauman et al. 2010; DiLeone and Ainsworth 2019). It is important to note that while some effects of cyanotoxins are broadly the same across multiple different groups, others are specific to just one. Identifying the ways that HABS affect the health of freshwater turtles is essential to understand the ways that certain taxa can resist or succumb to bloom toxins and, more critically, to mitigate or conserve threatened turtle species in areas where HABs are increasingly common.

### 3.3 Natural Water Quality Parameters

Water temperature affects dissolved oxygen levels, algal blooms, and the composition of microbiota communities, making it a common measure of water quality when paired with other natural properties (Meybeck et al. 2005). As ectothermic organisms, freshwater turtles are likewise influenced by water temperature (Boyer 1965). Many species bask as a form of behavioral thermoregulation, and alterations in this behavior may lead to increased threats from predators as turtles spend more time exposed (Ibáñez et al. 2015; Kashon and Carlson 2018). In bodies of water heated by discharge from a nuclear reactor, *T. scripta* only basked while partially submerged, while nearby populations in non‐thermal ponds basked totally out of water throughout the year, demonstrating a clear behavioral difference in warmer ambient temperatures (Spotila et al. 1984). Similarly, nocturnal basking was observed as a thermoregulatory behavior in tropical turtle populations when the temperature of the water was above their preferred temperature range and warmer than ambient air temperature (Nordberg and McKnight 2023). Experiments conducted on *Emydura macquarii* (Gray, 1831) also showed that individuals were more likely to bask when water temperatures were above their thermal preference (Kidman et al. 2023). Warming waters have also been associated with increased metabolic rates, oxygen consumption, and decreased dive times (Clark et al. 2008; Haskins and Tuberville 2022). As of yet, no peer‐reviewed studies have been conducted on the effects of cold‐water pollution on freshwater turtle behavior or physiology. However, in other ectothermic species such as fish, the growth of fry is considerably reduced in experimental studies replicating cold‐water pollution temperatures (Michie et al. 2020; Gilbert et al. 2024). Similar results were found when comparing the reproductive success of frogs in colder streams compared to those in warmer water; breeding activity, hatching, and metamorphosis occurred later in colder environments, and tadpoles were smaller compared to their warmer counterparts (Wheeler et al. 2015). These studies may provide a useful framework for understanding effects on freshwater turtles.

Freshwater habitats across the globe are becoming more saline as a result of mining, agricultural activity, and the dumping of industrial waste (Schuler et al. 2018; Iglesias 2020; Kaushal et al. 2021). A rapid and extreme increase in salinity concentrations of freshwater habitats may be detrimental to some species of turtles, while others may be better able to withstand the change. For example, a population of *Actinemys marmorata* (Baird & Girard 1852) living in coastal estuarine marshes was better able to maintain plasma osmolality at higher salinities than those from an inland freshwater stream, mainly via behavioral adjustments like reduced feeding and drinking (Agha et al. 2019). Another species, *M. terrapin* , is distributed almost exclusively in saltwater marshes, but hatchlings exposed to high salinity concentrations exhibited reduced growth, elevated heterophil: lymphocyte (H:L) ratios, and reduced food consumption (Ashley et al. 2021). Rising salinity levels may also lead to changes in population distributions, as salt‐intolerant species like *C. serpentina* (Linnaeus 1758), *Pseudemys gorzugi* (Ward, 1984), and *Clemmys guttata* (Schneider, 1792) move away from affected areas (Kinneary 1993; Agha et al. 2018; O'Dell et al. 2021; Mahan et al. 2022). The variations in physiological and behavioral plasticity of these species demonstrate how some turtle species and larger individuals within a species (Agha et al. 2018) more easily persist in freshwater habitats that are undergoing salinization, although long‐term survival may still pose a challenge even to robust populations (Herbert et al. 2015). As the effects of salinity on freshwater turtles have already been extensively reviewed (Agha et al. 2018), further review has not been included here.

Algal blooms, dams, and temperature can all lead to hypoxia in aquatic environments (Friedrich et al. 2014; Boyd 2020). While all freshwater turtles are air‐breathers, some species also possess the ability to draw dissolved oxygen from the water while submerged by absorbing O_2_ and releasing CO_2_ through the skin or the cloaca (Stone et al. 1992; FitzGibbon and Franklin 2010). As dissolved oxygen (DO) content declines, as in eutrophic conditions, bimodally respiring turtles may need to expend more time and energy foraging to compensate for their reduced diving ability (Schaffer et al. 2016). Bimodally respiring species, such as *Rheodytes leukops* (Legler & Cann, 1980), *Elseya irwinii* (Cann, 1997), and *Elusor macrurus* (Cann & Legler, 1994), had reduced dive times, depressed metabolic rates, and brachycardia when submerged in hypoxic conditions, indicating an inability to retrieve sufficient oxygen from the water to sustain longer dives under these conditions (Gordos et al. 2006; Clark et al. 2008; Schaffer et al. 2016). Similar effects were noted in *T. scripta* and *C. picta* , which brumate under water for weeks or months in anoxic conditions (Herbert and Jackson 1985). In hypoxic water, the metabolism and heart rate of *C. picta* decreased; this effect was particularly pronounced at a temperature of 3°C. These conditions are consistent with those under which *C. picta* brumates in its northern range, spending upwards of 125 days submerged while their heart rate and metabolism slows to reduce oxidative stress (Ultsch et al. 1999). In contrast, populations closer to the equator, which did not brumate, are unacclimated to hypoxic environments and less likely to survive long‐term exposure (Reese et al. 2004).

Freshwater turtles that breathe only air may be susceptible to the indirect effects of hypoxia; low DO conditions can reduce biomass and diversity of aquatic plants and insects, the primary food sources for many turtle species (Chambers et al. 2000). In riparian habitats, lower DO was associated with reduced turtle species abundance in both bimodally and non‐bimodally respiring turtle species (Dudley et al. 2015). However, this effect may have also been the result of other factors, such as a lack of food in low DO environments or a preference for denser aquatic vegetation which is associated with higher DO concentrations (Kofron and Schreiber 1985; Marchand and Litvaitis 2004; Caraco et al. 2006). Restored streams had higher chelonian species richness which, in addition to higher DO, also had greater cover in the lower canopy and higher primary production rates and a significantly different plant community than reference sites, all of which were correlated with the differences in turtle species abundance. This demonstrates both the far‐reaching importance of oxygen on the health of a freshwater ecosystem and the degree to which the lack of oxygen can affect air‐breathing aquatic fauna like freshwater turtles.

Information on the effects of nitrogen on population and behavior is lacking, but there is strong evidence that excess nitrogen can lead to poor health. Ambient exposure to ammonia resulted in oxidative stress, liver, and intestinal damage in the *Mauremys sinensis* (Gray, 1834) (Liang et al. 2020; Huang et al. 2021; Khan et al. 2021; Li et al. 2022). In addition, ammonia stress caused a significant shift in the composition of gut microbiota, decreasing the presence of beneficial bacteria and increasing that of pathogenic bacteria (Ding et al. 2021; Meng et al. 2023). Gut microbiota plays an important role in animal health and can assist with digestion and provide extra nutrients (Wexler 2007; Sehnal et al. 2021). The reduction of beneficial microbial diversity in response to high nitrogen and ammonia concentrations is therefore highly concerning. However, another study indicated that *Mauremys reevesii* , *Pelodiscus sinensis* , and *C. serpentina* were all able to tolerate ammonia exposure; *M. reevesii* and *P. sinensis* by increasing their oxygen carrying capability and *C. serpentina* by accelerating blood circulation and reducing blood viscosity (Chen et al. 2022). All three species also showed some signs of oxidative stress; however, they fully recovered after exposure. This demonstrates that some species may be able to withstand excess nitrogen levels in the short term, indicating at least some tolerance for eutrophication, which is often caused by a dramatic increase in nutrients like nitrogen (Wurtsbaugh et al. 2019).

Only two papers directly examined whether freshwater turtles were influenced by turbidity. The foraging ability of *C. picta* was tested in a laboratory setting under increasing turbidity; the outcome showed no effect of turbidity on the turtle's ability to locate and capture prey (Grosse et al. 2010). This result may be due to the generalist diet and feeding strategy of *C. picta* , which allows it to forage across a gradient of aquatic conditions more successfully and it would be valuable to test foraging ability in other species to determine how broadly this applies. In the more specialized, bimodally respiring *E. irwinii*, increased suspended solids had a clear negative effect on dive time, likely reducing the species' ability to utilize dissolved oxygen when submerged (Schaffer et al. 2016). This correlation raises concerns regarding the ability of bimodally respiring freshwater turtles to forage effectively, as turbidity in many areas is increasing due to agricultural runoff and artificial habitat modifications (Sherriff et al. 2015; Clermont et al. 2023). Species that rely on bimodal respiration for overwinter brumation may also be negatively impacted by rising turbidity levels, if it interferes with their ability to draw oxygen from the water (Jackson et al. 2004).

The number of highly acidic freshwater environments caused by drought (Li et al. 2017), mining (Qinggiang et al. 2022), or acid rain (Stoddard et al. 1999) is increasing across the globe. However, no papers to date have examined how pH directly affects turtles, although some included pH as a measured variable. Populations of *C. picta* occupied areas with higher pH, among other factors like permanence and vegetation (Cosentino et al. 2010). Other papers reported no correlation between freshwater turtle populations and pH; in many cases, species were in environments ranging from moderately basic (5.8–6.5) to neutral (7–8), indicating a tolerance for a range of pH concentrations (Roe et al. 2011; Yousefkhani et al. 2022). It is likely that different turtle species have a range of pH values in which they are tolerant. Other freshwater taxa, such as fish and amphibians, can tolerate some changes in pH, but increases in acidity may lead to reduced larval survival and growth, and reductions in ability to respond to olfactory cues (Serrano et al. 2016; Hasler et al. 2018). In areas where the threat of acidification is potentially or actually occurring, experimental studies to test whether turtles exhibit the same responses would be particularly beneficial.

The wide variation in the physiology and behavior of freshwater turtles, as well as the lack of data on many variables, makes it difficult to suggest any broad conclusions on how species might respond to changes in their habitat's water quality. Behavior, population dynamics, and life history are particularly unexplored, as are the effects of chemical factors like pH and nitrogen, both of which are naturally occurring but can be influenced by anthropogenic additions. Other impacts, such as cold‐water pollution (e.g., release of cold‐water from dams) and hypoxia from eutrophication, have only begun to be evaluated. In contrast, rising salinity concentrations do pose a threat to many freshwater turtle species (Agha et al. 2018).

### 3.4 Anthropogenic Pollutants

In addition to changes in naturally occurring water variables, anthropogenic pollutants occur in freshwater systems worldwide. Agricultural substances like pesticides and fertilizers, industrial and pharmaceutical chemicals, (polychlorinated biphenyls) PCBs, heavy metals, and sewage are harmful additives, and there are broad effects of these pollutants on aquatic organisms (Amoatey and Baawain 2019). In fish, accumulation of pollutants leads to immune suppression, reduced metabolism, and increased prevalence of disease (Malik et al. 2020). Similarly, buildup of microplastics and PCBs occurs in many bird species (de Kock and Randall 1984; Holland et al. 2016). Turtles, as largely permanent members of vulnerable freshwater ecosystems, are not exempt from exposure to these pollutants (Bodie and Semlitsch 2000; Bodie 2001).

Because of their long lifespan, freshwater turtles can be useful monitors for the buildup of heavy metals in their ecosystem (Moradi et al. 2022). Claw and soft tissue samples are accurate indicators of environmental metal contamination, as are DNA samples for radionuclides (Martínez‐López et al. 2017; Slimani et al. 2018). Older turtles tend to have higher concentrations of metals than younger individuals, indicating that they may accumulate contaminants over time (Beau et al. 2019). Heavy metals build up through trophic transfer, and prey such as invertebrates and small fish are more likely to have higher metal concentrations than algae and plant material; these higher concentrations can then transfer up the food web into higher trophic levels, like turtles (Liu et al. 2019). Heavy metals can also be transferred from a female turtle to her eggs; mercury and copper were both detected in *T. scripta* and *C. serpentina* eggs as a result of maternal transfer, though concentrations in embryos were lower than in tissue from the corresponding mother (Yu et al. 2011; Hopkins et al. 2013). Because these contaminants can be transferred to offspring from the mother, future use of turtles to track heavy metals should continue to consider multiple life stages, including eggs and hatchlings, to establish a baseline concentration.

Physiologically, exposure to heavy metals, like mercury and lead, is associated with low body condition, high H:L ratios, and increased oxidative stress in *M. leprosa* and *T. scripta* at sites contaminated with chromium, copper, mercury, and lead (Yu et al. 2011; Ortiz‐Santaliestra et al. 2019). Mercury specifically was also correlated with lower rates of infertility and low embryonic survival in *C. serpentina* , and long‐term exposure was associated with low sperm count in males and reduced gonad weight in *C. picta* adults (Yu et al. 2011; Hopkins et al. 2013).

As with heavy metals, accumulation of pesticides over time is reflected in samples of claw and soft tissue, making freshwater turtles useful indicators of both current and historical agricultural chemicals (Moss et al. 2009; Douros et al. 2015; Meyer et al. 2016). Diet may also affect the blood concentration levels of pesticides; *Sternotherus odoratus* (Latreille, 1801), a bottom‐dwelling omnivore, had higher concentrations of dichlorodiphenyltrichloroethane (DDT) than *T. scripta* , which is carnivorous as a juvenile and herbivorous as an adult (Moss et al. 2009). While the long‐term health effects of these chemicals are unclear, short‐term exposure to herbicides causes oxidative stress and lowers gut microbial diversity in turtles (Héritier et al. 2017; Madison et al. 2018). However, immunity does not seem to be immediately affected by chemical exposure; a common herbicide was not correlated with ranavirus susceptibility, though only 57.7% of *T. scripta* exposed to ranavirus were infected, and the study recorded low growth rates for all groups (Goodman et al. 2021). Exposure to agricultural chemicals, particularly pesticides, was correlated with larger females and higher body condition in a site downstream of a wastewater plant compared to an uncontaminated upstream site; however, the population exposed to agricultural chemicals was also far more male‐biased than the uncontaminated population, which may indicate an effect of pesticides on the temperature‐dependent sex determination of embryos (Le Gal et al. 2023). However, a higher body condition is not indicative of health; indeed, larger individuals tend to have poorer immune responses (Polo‐Cavia et al. 2010), and females that invest heavily in reproduction by producing larger or heavier clutches have much higher stress levels (Rafferty et al. 2014). Over time, exposure to these chemicals may therefore decrease the overall fitness of populations in contaminated habitats.

The information available on polychlorinated biphenyls was somewhat limited in scope; most papers focused on *C. serpentina* as a study species, and experimental trials were largely performed on juveniles. *C. serpentina* exposed via maternal transfer to PCBs had a reduced growth and significantly higher mortality rate at 8 months old than those from a reference site (Eisenreich et al. 2009). Reduced growth, as well as decreased metabolic rates and an altered respiratory pattern was also observed in juveniles exposed via aquatic contamination (Holliday et al. 2009). Both eggs and tissues from adults had elevated PCB levels at contaminated sites and, as with other pollutants, PCBs appeared to accumulate with age (Hebert et al. 1993; Ashpole et al. 2004; Burkart et al. 2021). However, there are few studies that indicate any long‐term health, behavioral, or population effects of this accumulation. In a PCB contaminated river, individuals were heavier than those from a reference site, but no other correlations with relative abundance, age, sex, or body condition were detected (Gibbs et al. 2017).

In the Anthropocene era, many habitats commonly used by freshwater turtles, such as lakes and rivers, are increasingly contaminated by urban and industrial pollution (Mushtaq et al. 2020). Some species have adapted well to these environments; populations of *M. leprosa* , *A. marmorata* , and *Phrynops geoffroanus* (Schweigger, 1812) live in degraded habitats, such as sewage systems, pollution control plants, and urban rivers (Souza and Abe 2000; Polo‐Cavia et al. 2010; El Hassani et al. 2019). In the case of *M. leprosa* and *P. geoffroanus* , population density and an individual's size and body condition were all higher in sewers compared to a reference site, likely due to the abundance of food and absence of predators (El Hassani et al. 2019). Likewise, individual *A. marmorata* inhabiting a pollution control plant were larger and had a higher body condition compared to an uncontaminated reference site (Polo‐Cavia et al. 2010). However, *A. marmorata* also had a higher H:L ratio and lower immune responses in the sewage plant, indicating physiological stress and poor health. Long‐term effects of these responses should therefore be studied closely, as prolonged stress may exacerbate disease and parasitization, and some industrial chemicals can even disrupt development and reproductive capabilities (Siegel 1980; Guillette and Gunderson 2001; Hing et al. 2016). In some areas, these effects are already becoming noticeable; populations of *Graptemys flavimaculata* (Cagle, 1954) have steadily declined over 11 years as a result of pollution from a wood pulp processing facility (Shelby and Mendonça 2001). This decline is likely due to an increase in embryonic mortality and impaired reproductive capabilities due to decreased plasma testosterone and increased estrogen in males of the species, attributed to 2,3,7,8‐tetrachlorodibenzo‐p‐dioxin (TCDD) contamination from the wood pulp mill. TCDD can disrupt endocrine functioning and development in many vertebrates and increases mortality in exposed freshwater turtle embryos (Doering et al. 2022).

In addition to long‐term impacts on populations, more immediate effects of exposure to urban and industrial chemicals occur. A laboratory study found that exposing *P. geoffroanus* to benzo[a]pyrene, a chemical often found in urban and industrial wastewater, led to oxidative stress and blood cell DNA damage (Silva et al. 2016). Likewise, neonate *Podocnemis expansa* (Schweigger, 1812) exposed to antineoplastic drugs presented with a number of red blood cell abnormalities, any of which can lead to cell death, genomic instability, or to cancer (Mesak et al. 2019). However, they also demonstrated a remarkable ability to recover, reducing the number of abnormalities once removed to an enclosure with uncontaminated water. Future studies should continue to investigate the ability of turtles to recover from exposure to potentially damaging chemicals, as well as continuing to monitor turtles in situ and experimentally to record any long‐term effects.

### 3.5 Oil & Coal Spills

Though oil and coal spills are more commonly associated with marine environments, both occur frequently in freshwater habitats, leading to extensive damage including impaired health and reproductive output, large die‐offs, decreases in biodiversity among fish, birds, and mammals (Steen et al. 1999; Azevedo‐Santos et al. 2016). The same effects hold true for freshwater turtles. As a result of oil spills, freshwater turtle populations in the rivers and wetlands in the United States experienced mortalities of roughly 6%, likely due to direct exposure and ingestion (Saba and Spotila 2003; Otten et al. 2022).

More broadly, an oil spill in Nigeria led to significantly diminished turtle species diversity and richness, decreasing from four freshwater turtle species to two (Luiselli and Akani 2003). Additionally, the species that remained, *Pelusios castaneus* (Schweigger, 1812) and *Pelusios niger* (Duméril & Bibron, 1835), exhibited a marked reduction in the variety of their diet as their preferred prey disappeared from the affected area (Luiselli et al. 2006). This study demonstrates the potential for flow‐on indirect effects from contaminated sites and the importance of longer‐term monitoring to understand such impacts.

Like oil spills, contamination from coal and coal ash leads to contamination of freshwater habitats and mass die‐offs of fish due to methylmercury poisoning (Ruhl et al. 2009). Mercury exposure is associated with decreased reproductive and hatching success in freshwater turtles (Hopkins et al. 2013). However, there is little evidence that coal ash pollution has any negative impacts on freshwater turtles. Among *T. scripta* and *C. serpentina* exposed to a coal‐fly ash spill, trace amounts of mercury, as well as arsenic and selenium, were detected in both adults and eggs laid by captured gravid females, though in relatively low concentrations. Additionally, no detrimental health or reproductive effects were documented in either species (Steen et al. 1999; Van Dyke et al. 2014, 2017). However, all three studies were conducted several years after the spill, so any direct impacts of coal ash spills remain undetermined. In the face of the overwhelming evidence of detrimental effects of mercury poisoning on freshwater turtle fitness and reproduction, the effects of coal and coal by‐product contamination on freshwater turtles should continue to be examined closely in both the immediate aftermath and the long term (Meyer et al. 2014; Thompson et al. 2018; Beau et al. 2019). Due to the ethical and practical difficulties of studying the effects of exposure to coal and oil on freshwater turtles proactively or experimentally, it may be difficult to expand this area of research. However, given the large capacity and historic pollution effect of these industries along with the large revenue that they produce, more investment into understanding their potential impacts and knowledge of how to mitigate exposure would be beneficial.

### 3.6 Future Directions

Comprehensively understanding how freshwater turtles are affected by their freshwater environment is an ongoing challenge in ecology and conservation. Species classified as Least Concern are better understood specifically because of their status as common species, they are easy to obtain for controlled environment experiments and are frequently encountered in the wild. *T. scripta* , the most well studied species, has been introduced on every continent except Antarctica, and it is considered an invasive species in most areas outside its native range (Ramsay et al. 2007; Ferronato et al. 2009; Martins et al. 2014; Standfuss et al. 2016; Mo 2019). This presents an opportunity to better understand comparative regional impacts to native turtles by providing a standardized model turtle throughout the world.

In contrast, effects of water quality parameters on Critically Endangered species are poorly known. Rarity makes such threatened species difficult to study in the wild, and using them for experiments which might expose them to hazardous substances like sewage or industrial pollutants is counterintuitive to conservation programs. For anthropogenic‐induced variables such as pesticides or oil spills, model species may be much more suitable both to minimize the risk of harming endangered native species and because studies across many unrelated species have already indicated that the effects of exposure to these variables may be similar. However, where large numbers of threatened species' offspring are produced such as in farms or with captive stock, there may be opportunity to pair captive bred turtles with scientific research to better understand how currently unknown water quality variables affect these species.

While using model species like *T. scripta* as a substitute for more critically endangered species can be useful, special care should also be taken not apply the results to all chelonians without further research, especially with regards to naturally occurring aquatic characteristics like salinity and dissolved oxygen. Many turtle species have different sensitivities to different parameters, and even populations of the same species living in different localities may have vastly different responses to the same variable (e.g., Agha et al. 2019). For these naturally occurring variables, more nuanced understandings across taxa is required.

The other risk of substituting a model species for more endangered native species is that of creating an artificial gap in knowledge. For example, of the 25 papers that were published in China, 14 were on the invasive *T. scripta* , and a further 10 were on the commercially available Chinese species, *M. sinensis* and *P. sinensis* . This oversight is tragic considering that China is home to 29 endemic species of freshwater turtles and tortoises (Rhodin et al. 2025), most of which are threatened to some degree (Wu et al. 2020) including, ironically, wild populations of *M. sinensis* (Li et al. 2021). In the case of *M. sinensis* , all of the papers which included this species as a subject were controlled lab experiments investigating physiological responses to specific pollutants; there were no papers that examined how individuals respond to any environmental variables in situ. In regions like this, that are both rich in chelonian taxa and undergoing rapid declines or decimation of natural habitats (He et al. 2014), researchers must exercise extra caution not to let use of model species take priority over the study and conservation of endemics, or the use of captive species to overtake study of populations as they occur in the wild.

Another difficulty in studying turtles is the mechanism through which research is undertaken. Some variables, such as salinity or pH, are usefully manipulated in replicated laboratory or correlative field studies to understand physiological or behavioral responses. However, understanding the longer‐term responses of sublethal effects, such as differences in prey in response to changes in water quality, and manipulating more complex variables like oil spills and harmful algal blooms, is more challenging and reflects the paucity of experimental understanding in the literature (Otten et al. 2022). Compounding the issue further, it can be difficult to draw meaningful conclusions from studies in situ, as there are often multiple variables interacting in natural environments which can make interpretation difficult. For example, the dissolved oxygen content in a pond can be increased or decreased by the presence of plankton, aquatic vegetation, and fish and aquatic vertebrates, all of which could have independent effects on turtle abundance, health, or reproductive habits (Ryan et al. 2015). Even simple factors like specific water body attributes can influence the physiology of turtle populations in a small area (Scheelings 2023). Future studies should therefore restrain themselves from focusing on only one aspect of water quality whenever possible. Efforts should also be made to study freshwater ecosystems for longer periods of time, as many watersheds cycle through seasonal periods of flooding and drought or extreme temperature changes. Long term studies are incredibly important for understanding the fluctuations of health and population dynamics, and pairing them with long‐term monitoring of water quality and environmental contaminants should be a priority when considering whether large changes will have a significant impact on species.

In situ studies need to be complemented with experimental research; in a controlled environment where environmental factors can be mitigated or eliminated entirely. However, this approach poses its own challenges. The ethics of subjecting animals, particularly threatened species, to potentially hazardous conditions, even in the name of future conservation, requires strong ethical justification. Moreover, some factors such as industrial pollution or sewage systems may be difficult to simulate in a laboratory environment. In many cases, water must be brought in from an external source, a process which can be both costly and time consuming (Mahmood et al. 2005; Piazza et al. 2019). Some experimental studies on fish have had success isolating and manipulating pre‐existing bodies of water or building cages in which fish can be held for long periods of time (Cazenave et al. 2014; Piazza et al. 2019). However, due to the mobile nature of freshwater turtles, some of which may aestivate terrestrially away from water sources due to drought or lack of food, isolating them in their natural environments may be difficult (Roe and Georges 2008; Welty et al. 2013). These challenges make the contributions from controlled experiments all the more important and emphasize the need to pair studies in controlled environments with more naturalistic studies.

The vast majority of papers on water quality effects on turtles examined the direct effects of various aquatic characteristics on turtle physiology, behavior, reproduction, survival, and population demographics. There is, however, another aspect which was not included: indirect effects. Turtles are not isolated from the ecosystem in which they live; they affect and are affected by a variety of biotic and abiotic factors which may be in turn influenced by the water quality of the environment. For example, while *C. longicollis* itself displays some resilience for high salinity conditions, those same conditions can provide an ideal breeding ground for tube worms which can parasitize turtles, causing high mortalities (Bower et al. 2012). There are also indications that turtles living in more urban areas may be at greater risk of being parasitized by leeches and the hemoparasites they carry, as the organic pollutants in these areas provide a more favorable habitat for leeches (de Campos Brites and Rantin 2004). These indirect effects, as well as those that may affect the abundance and composition of animals and plants that turtles feed on, the structures on which they bask, and the abundance and composition of turtle predators, are important areas of study and should not be overlooked.

## 4 Conclusion

1. There are opportunities to use invasive or common species as a model for more potentially harmful variables, such as exposure to pesticides or oil spills, although the results of these studies should only be applied broadly with extreme caution. Both proactive research (i.e., controlled experiments) and reactive (in situ) research is required for a holistic understanding of how turtles interact and respond to their aquatic environment. In addition, special care should be taken to study threatened native species whenever possible, as they are more likely to be negatively impacted by changes in their environment.
2. Many common species of freshwater turtle are resilient, able to survive in sewage systems or in estuarine wetlands that are becoming increasingly saline because of rising sea levels. Some species can also tolerate exposure to contaminants that are harmful to other taxa and may be used as environmental monitors for pollution. Given this resiliency, changes to a species' natural aquatic environment may not be the greatest threat to their survival. However, given the large number of endangered freshwater turtle species, particularly those that are habituated to specific conditions, this ability to persevere cannot be taken for granted.
3. There are still innumerable deficits in our understanding of chelonian responses to changing aquatic environments, particularly among those species which are most critically endangered. Filling those gaps is of vital importance if we are to preserve these remarkable, oft‐overlooked animals for future generations.

## Author Contributions

**K. Sullivan:** conceptualization (equal), data curation (equal), formal analysis (equal), investigation (equal), writing – original draft (equal), writing – review and editing (equal). **Deb Bower:** conceptualization (equal), funding acquisition (equal), project administration (equal), supervision (equal), writing – review and editing (equal). **Eric Nordberg:** conceptualization (equal), funding acquisition (equal), project administration (equal), supervision (equal), writing – review and editing (equal).

## Funding

All funding for this article was provided by the University of New England, Australia.

## Conflicts of Interest

The authors declare no conflicts of interest.

## Supporting information


**Data S1:** Lit review all papers.

## Data Availability

The authors confirm that the data supporting the findings of this study are available within the article and Data S1.
